# Association of Concurrent Olfactory Dysfunction and Probable Rapid Eye Movement Sleep Behavior Disorder with Early Parkinson's Disease Progression

**DOI:** 10.1002/mdc3.13511

**Published:** 2022-07-22

**Authors:** Ying Chen, Nai‐Jia Xue, Yi Fang, Chong‐Yao Jin, Yao‐Lin Li, Jun Tian, Ya‐Ping Yan, Xin‐Zhen Yin, Bao‐Rong Zhang, Jia‐Li Pu

**Affiliations:** ^1^ Department of Neurology, Second Affiliated Hospital, School of Medicine Zhejiang University Zhejiang China

**Keywords:** Parkinson's disease, REM sleep behavior disorder, olfactory dysfunction, motor progression, cognitive function

## Abstract

**Background:**

Parkinson's disease (PD), with either rapid eye movement sleep behavior disorder (RBD) or olfactory dysfunction (OD), has been associated with disease progression. However, there is currently heterogeneity in predicting prognosis.

**Objectives:**

To identify whether the concurrent presence of OD and probable RBD (pRBD) in PD (Dual hit in PD, PD‐DH) is associated with disease progression.

**Methods:**

We included 420 patients with de novo PD from the Parkinson's Progression Markers Initiative: 180 PD only (PD), 82 PD with OD (PD‐OD), 94 PD with pRBD (PD‐pRBD), and 64 PD with both OD and pRBD (PD‐DH). Participants underwent motor and nonmotor evaluations, dopamine transporter imaging, and cerebrospinal fluid (CSF) assessment. Data were analyzed with generalized estimating equations and Cox proportional hazards analysis.

**Results:**

The PD‐DH subtype was associated with higher scores and faster progression rates in Movement Disorder Society–Unified PD Rating Scale (MDS‐UPDRS) Parts II and III. Also, patients in PD‐DH group had faster deterioration in nonmotor symptoms, including MDS‐UPDRS Part I score, Montreal Cognitive Assessment, Hopkins Verbal Learning Test–Revised, Wechsler Memory Scale‐Third edition (WMS‐III) Letter Number Sequencing score, Symbol Digit Modalities Test, and Scales for Outcomes in PD–Autonomic scores, with all *P* values <0.002. Moreover, the PD‐DH subtype had a higher mild cognitive impairment risk (hazard ratio = 1.756, 95% confidence interval [CI] = 1.132–2.722; *P* = 0.012), faster decline in caudate standard uptake values (β = −0.03, 95% CI = −0.06 to −0.008, *P* = 0.012), and CSF α‐synuclein levels (β = −77, 95% CI = −149 to −5, *P* = 0.034) than the PD group.

**Conclusion:**

Coexisting pRBD and OD in patients with PD may be associated with faster progressions in motor measurements and in cognitive and autonomic symptoms, indicating PD‐DH as a more aggressive subtype for PD.

Parkinson's disease (PD) is the second most common neurodegenerative disease and is characterized by progressive degeneration of dopaminergic substantia nigra neurons and the formation of pathological α‐synuclein (α‐syn) aggregates. Accumulating evidence indicates that PD varies in its clinical manifestations and prognosis.^1^ Accordingly, identifying PD subtypes may help us understand the disease's mechanisms, predict prognosis, and design better clinical trials. However, to date, no specific PD subtype that can accurately predict disease progression has been identified.

Idiopathic rapid eye movement sleep behavior disorder (RBD), characterized by dream‐enacting behaviors, is considered the most reliable and powerful prodromal marker of PD, occurring in 35% to 60% of patients with PD.^2^ Although increasing evidence shows that PD with RBD is associated with a higher nonmotor symptom burden and faster motor progression,^3^, ^4^, ^5^, ^6^ there have been some inconsistent results in recent years.^7^, ^8^, ^9^ Liu et al found that patients with PD and RBD do not exhibit faster increase in motor deficits.^8^ Hogue et al showed that the presence of RBD was not significantly associated with cognitive decline.^9^ Moreover, Pagano et al reported that RBD in early PD was associated with faster motor progression in patients with greater α‐syn and dopaminergic pathology and with faster cognitive decline in patients with greater α‐syn and amyloid pathology.^7^ Therefore, the accelerated PD progression attributed to RBD is still controversial; whether other factors influence the rate of disease progression in the presence of PD with RBD needs to be characterized.

Olfactory dysfunction (OD), another important prodromal sign of PD, was previously associated with early disease conversion from RBD to PD in a large cohort study.^10^ OD was also a reportedly useful predictor of disease progression.^11^, ^12^, ^13^, ^14^ Data on the relationship between OD, cognitive impairment, and motor symptoms in patients with PD are contradictory.^15^, ^16^ Doty et al suggested that olfactory deficits are not correlated with cognitive abilities and motor symptoms.^15^ Similarly, Rossi et al indicated that OD in early PD was not associated with motor dysfunction.^16^

So far, there is controversy on the role of OD or RBD as single factors influencing PD progression, and it is still unclear if there is an additive effect between these two markers. Thus, in this study, we sought to identify a more accurate PD subtype according to the presence of both RBD and OD and evaluate its effect on disease progression and the differences in effects from each single factor.

## Methods

### Study Design and Participants

The Parkinson's Progression Markers Initiative (PPMI) is an ongoing, longitudinal, multicenter, observational, clinical study aiming to identify biomarkers for predicting PD progression.^17^ This longitudinal study included 421 drug‐naïve patients with PD included in the PPMI database (www.ppmi-info.org) until the end of March 2021. The inclusion criteria were diagnosis with PD within 2 years and baseline Hoehn and Yahr stage of I–II. To avoid the effect of chronicity on PD manifestations, PD duration was calculated based on age at onset and follow‐up time.

All participating institutions' regional ethical committees approved the PPMI, and all study participants provided written informed consent. This study was approved by the institutional review board at the PPMI site.

### Clinical Assessments

The clinical data were obtained at baseline and at the 12‐, 24‐, 36‐, 48,‐ and 60‐month follow‐ups (mean, 4.32 ± 1.36 years). For the Movement Disorder Society–sponsored revision of the Unified Parkinson's Disease Rating Scale (MDS‐UPDRS) scores, we extended the follow‐up by including the measurements at 72, 84, and 96 months (mean follow‐up, 5.42 ± 2.54 years). The severity of motor symptoms was assessed using the MDS‐UPDRS Part II (motor aspects of experiences of daily living) and Part III (motor examinations) scores in the *off* medication state.

We applied the MDS‐UPDRS Part I score for the evaluation of overall nonmotor symptoms. Also, detailed nonmotor symptoms including autonomic dysfunctions and cognitive deficits were also included in our analysis. The Scales for Outcomes in Parkinson's Disease–Autonomic (Scopa‐AUT) was used to evaluate autonomic dysfunction, and the Montreal Cognitive Assessment (MoCA) was used to assess global cognitive status. Furthermore, verbal memory assessments were conducted by using domain‐specific neuropsychological performance tools, including the Hopkins Verbal Learning Test–Revised (HVLT‐R), which evaluates immediate recall, delayed recall, and delayed recognition and retention. The Semantic Fluency test (SFT) was used for measuring executive function, the Wechsler Memory Scale‐Third edition (WMS‐III) Letter Number Sequencing (LNS) test for working memory, and the Symbol Digit Modalities Test (SDMT) for attention function. Mild cognitive impairment (MCI) was defined as ≥2 cognitive test scores >1.5 standard deviation below the standardized mean.^18^

The presence of probable RBD (pRBD) was assessed by the RBD Screening Questionnaire using a cutoff of 5 points as previously reported.^7^, ^19^ Olfactory function was assessed via the University of Pennsylvania Smell Identification Test, and OD was defined as anosmia using a cutoff of 18 points according to previous studies.^20^ Based on the existence of either pRBD or OD, patients with PD were assigned to the following 4 groups: (1) group I, PD; (2) group II, PD only with olfactory dysfunction (PD‐OD); group III, PD only with pRBD (PD‐pRBD); and (4) group IV, PD with both OD and pRBD (Dual hit in PD, PD‐DH).^7^, ^21^

### Cerebrospinal Fluid Biomarkers

Cerebrospinal fluid (CSF) samples were obtained from patients with PD at baseline and at the 12‐, 24‐, 36‐month visits. CSF amyloid‐β1–42 (Aβ42), total tau (t‐tau), and phosphorylated tau at threonine 181 (P‐tau) were measured using INNO‐BIA AlzBio3 (Ghent, Belgium) immunoassay kit‐based reagents. CSF α‐syn was measured via an enzyme‐linked immunosorbent assay.^22^

### Single‐Photon Emission Computed Tomography Dopaminergic Imaging

Single‐photon emission computed tomography (SPECT) with a dopamine transporter (DAT) tracer (Ioflupane I‐123, Iodine 123‐radiolabeled 2β‐carbomethoxy‐3β‐(4‐iodophenyl)‐N‐(3‐fluoropropyl) nortropaneI [^123^I‐FP‐CIT], DaTSCAN) was performed during screening and at baseline and at the 12‐, 24‐, and 48‐month visits using a standardized protocol (http://www.ppmi-info.org/data). The regions of interest were set as the left and right caudate, putamen, and the occipital region (reference tissue). The specific binding ratios (SBRs) were calculated as (target region/reference region) − 1. The mean values of the more‐affected sides were used for analysis.

### Statistical Analysis

Continuous variables are presented as mean ± SD, and categorical variables are presented as counts. All variable distributions were tested for variance homogeneity and normality using the Kolmogorov–Smirnov test. Continuous variables (parametric variables) were compared via 1‐way analysis of variance (with Bonferroni post hoc test), whereas categorical variables were compared using the χ^2^ test. Nonparametric variables were compared using Kruskal–Wallis tests.

Disease progression was analyzed using each patient's data for 5 years beginning at diagnosis, and for MDS‐UPDRS scores we further extended the follow‐up time to 8 years. However, the number of follow‐up visits and intervals varied among the patients, and some data were missing. Therefore, we selected generalized estimating equations (GEEs) for a more comprehensive longitudinal comparison of subtypes. For each measurement, PD subtype and visit time were set as the main independent variables in GEEs to compare the group difference, with age, age at onset, sex, genetic information of Glucosylceramidase Beta (*GBA*) and Leucine Rich Repeat Kinase 2 (*LRRK2*), and each baseline score adjusted as potential covariates in each model. Furthermore, we included group × time interactions in GEEs to evaluate the differences in the clinical measurement scores among the 4 groups in a unit of time (year) during follow‐up, with the same corrected covariates noted previously. We also added levodopa dose as a covariate in the analysis of motor symptoms. In addition, we added educational attainment and genetic information of Apolipoprotein E4 (*ApoE4*) for cognitive function analysis. We first set the other PD subtypes as the reference group, followed by PD‐DH. Cox proportional hazards analysis was used to investigate pRBD and OD as predictors of MCI after correction for the covariates listed previously. All statistical analyses were performed using SPSS software (version 20.0; IBM Corp., Armonk, NY) and GraphPad Prism (version 8.3; GraphPad Software, La Jolla, CA). All statistical tests were 2‐tailed, and *P* values <0.05 were considered significant.

### Data Sharing

The data that support the findings of this study are openly available from at https://www.ppmi-info.org/.

## Results

### Baseline Demographics and Clinical Characteristics

A total of 420 patients were included: 180 (42.9%) with PD, 64 (15.2%) with PD‐DH, 82 (19.5%) with PD‐OD, and 94 (22.4%) with PD‐pRBD. Baseline demographic characteristics are listed in Table 1. Patients in the PD‐OD and PD‐DH groups tended to be older and have a later age of onset (*P* < 0.001). There was no significant difference in disease duration, pathologic *GBA* or *LRRK2* variant status, Hoehn and Yahr stage, and MDS‐UPDRS Part III score among the 4 groups at baseline. For nonmotor symptoms, patients in PD‐DH group exhibited the highest Scopa‐AUT scores (*P* < 0.001) and the lowest Benton Judgment of Line Orientation, SDMT, and SFT scores (all *P* < 0.05) among the 4 groups. The PD‐pRBD group showed higher MDS‐UPDRS Part I, MDS‐UPDRS Part II, and MDS‐UPDRS total scores as well as autonomic dysfunction scores than the PD group. Further evaluation of the differences in DAT and CSF protein levels at baseline indicated that only the PD‐OD group had a larger dopaminergic deficit in the caudate (*P* = 0.007 and *P* = 0.021) and putamen (*P* = 0.024 and *P* = 0.011) than the PD group, as shown in Table 2.

**TABLE 1:** Baseline demographics and clinical characteristics

Characteristics	Group I PD	Group II PD‐OD	Group III PD‐RBD	Group IV PD‐DH	*P* Value	Post Hoc *P* Value
Sample size, n	180	82	94	64	–	–
Age, y	59.6 (9.7)	65.5 (8.4)	60.2 (10.3)	64.4 (7.9)	<0.001^a^	I vs. II, I vs. IV, II vs. III
Sex, male/female	106/74	59/23	58/36	52/12	0.006^b^	–
Age onset, y	57.4 (10.0)	63.8 (8.9)	58.3 (10.3)	62.4 (7.9)	<0.001^a^	I vs. II, I vs. IV, II vs. III
Duration, mo	6.9 (6.4)	6.3 (6.8)	6.2 (6.2)	6.8 (6.6)	0.588^c^	–
Pathologic variants						
*GBA*	18/162	9/73	17/77	8/56	0.271	–
*LRRK2*	58/117	28/50	24/69	13/50	0.138	–
H&Y stage, 1/2/3	77/93/2	30/49/0	43/46/0	25/36/0	0.562^b^	–
MDS‐UPDRS Part III	19.8 (8.2)	22.6 (9.6)	20.1 (8.4)	22.0 (9.2)	0.155^c^	–
MDS‐UPDRS Part I	4.2 (2.9)	5.5 (4.2)	7.2 (4.9)	6.7 (3.8)	<0.001^a^	I vs. III, I vs. IV
MDS‐UPDRS Part II	4.8 (3.6)	5.5 (3.5)	7.1 (4.9)	7.2 (4.4)	<0.001^a^	I vs. III, I vs. IV
MDS‐UPDRS total	28.9 (11.0)	33.7 (13.6)	34.5 (14.5)	36.0 (13.8)	<0.001^c^	I vs. III, I vs. IV
MoCA	27.4(2.0)	26.7 (2.7)	27.2 (2.3)	26.6 (2.3)	0.052^a^	–
HVLT‐R total recall, total score	46.6 (10.6)	46.3 (10.7)	44.8 (10.6)	43.2 (10.9)	0.131c	
Delayed recall	46.3 (11.0)	44.9 (10.5)	43.1 (11.3)	43.5 (10.2)	0.052^a^	
Retention	48.3 (11.5)	46.6 (11.2)	45.5 (11.8)	47.1 (10.1)	0.116^a^	
Recognition	46.2 (11.1)	44.1 (12.2)	44.1 (10.5)	42.9 (11.5)	0.151^a^	
LNS	11.0 (2.5)	10.1 (2.8)	10.3 (2.3)	10.1 (2.9)	0.008^a^	I vs. II
SDMT	43.4 (9.2)	39.3 (9.7)	40.8 (9.0)	37.6 (10.5)	<0.001^a^	I vs. II, I vs. IV
SFT	50.8 (10.9)	47.2 (12.6)	48.9 (11.6)	44.2 (10.7)	<0.001^a^	I vs. IV, I vs. II
Scopa‐AUT	7.3 (4.8)	9.4 (5.8)	11.0 (6.5)	13.4 (7.0)	<0.001^a^	I vs. III, I vs. IV, II vs. IV
Gastrointestinal	1.5 (1.7)	2.1 (2.1)	2.6 (2.1)	3.3 (2.1)	<0.001^a^	I vs. III, I vs. IV, II vs. IV
Urinary	3.3 (2.3)	4.5 (2.9)	4.6 (3.1)	5.5 (3.8)	<0.001^a^	I vs. II, I vs. III, I vs. IV
Cardiovascular	0.3 (0.6)	0.3 (0.5)	0.7 (0.9)	0.5 (0.9)	<0.001^a^	I vs. III, II vs. III

^a^ Kruskal–Wallis test.

^b^ χ^2^ test.

^c^ *P* values are calculated using 1‐way analysis of variance.

Data was shown as mean(SD). Abbreviations: PD, Parkinson's disease; OD, olfactory dysfunction; RBD, rapid eye movement sleep behavior disorder; DH, dual hit; GBA, glucosylceramiddase beta; LRRK2, leuine rich repeat kinase 2; H&Y, Hoehn and Yahr; MDS, Movement Disorder Society; UPDRS, Unified Parkinson's Disease Rating Scale; MoCA, Montreal Cognitive Assessment; HVLT‐R, Hopkins Verbal Learning Test–Revised; LNS, Wechsler Memory Scale‐Third edition (WMS‐III) Letter Number Sequencing; SDMT, Symbol Digit Modalities Test; SFT, Semantic Verbal Language Fluency Test; Scopa‐AUT, Scales for Outcomes in Parkinson's Disease–Autonomic.

**TABLE 2:** Baseline CSF and DAT imaging pathology

Outcome	PD	PD‐OD	PD‐RBD	PD‐DH	*P* Value	Post Hoc *P* Value
DAT imaging						
Low putamen	0.69 (0.25)	0.61 (0.22)	0.68 (0.32)	0.64 (0.22)	0.024^a^	I vs. II
Low caudate	1.88 (0.51)	1.66 (0.52)	1.84 (0.60)	1.75 (0.51)	0.007^a^	I vs. II
Mean putamen	0.86 (0.28)	0.75 (0.22)	0.85 (0.37)	0.78 (0.27)	0.011^a^	I vs. II
Mean caudate	2.06 (0.52)	1.85 (0.51)	2.02 (0.62)	1.91 (0.55)	0.021^b^	I vs. II
CSF, markers, pg/mL						
α‐syn	1465 (620)	1574 (720)	1444 (632)	1542 (725)	0.810^a^	–
Aβ42	899 (369)	885 (405)	954 (481)	911 (427)	0.740^a^	–
Tau	159 (51)	167 (66)	167 (61)	180 (69)	0.161^a^	–
P‐tau	13.4 (5.0)	14.0 (6.5)	13.8 (6.1)	15.3 (6.7)	0.294^a^	–

^a^ Kruskal–Wallis test.

^b^ *P* values are calculated using 1‐way analysis of variance.

Data was shown as mean (SD). Abbreviations: CSF, cerebrospinal fluid; DAT, dopamine transporter; PD, Parkinson's disease; OD, olfactory dysfunction; RBD, rapid eye movement sleep behavior disorder; DH, dual hit; α‐syn, α‐synuclein; Aβ42, β‐amyloid 1–42; P‐tau, phosphorylated tau at threonine 181.

### Longitudinal Changes of Motor and Nonmotor Symptoms

Significant group differences regarding an average measurement over all time points during follow‐up were observed in motor and nonmotor symptoms (Table 3). Patients in the PD‐DH group exhibited the highest increment in MDS‐UPDRS Part II and Part III scores (2.2 and 1.9 points more than the PD group, with *P* < 0.001 and *P* = 0.040, respectively). For overall nonmotor symptoms, patients in the PD‐DH, PD‐pRBD, and PD‐OD groups had higher scores on the MDS‐UPDRS Part I (2.6, 1.9, and 0.8 points more increase compared with the PD group, respectively), but the highest increment was also found in the PD‐DH group (2.6 points, *P* < 0.001). Similar remarked progressions could be also observed in several cognition measurements, namely MoCA (1.0 points lower in PD‐DH, 0.6 points lower in PD‐pRBD, and 0.4 points lower in PD‐OD, with *P* = 0.001, 0.003, and 0.068, respectively), HVLT‐R total recall (4.8, 1.6, and 2.9 points lower), LNS (0.8, 0.4, and 0.3 points lower), and SDMT (3.2, 1.9, and 1.7 points lower). Regarding the autonomic function, the patients in the PD‐DH and PD‐pRBD groups exhibited significant higher Scopa‐AUT scores than those in the PD group (2.1 and 1.1 points more, respectively), whereas no significant difference was observed between the PD‐OD and PD groups.

**TABLE 3:** Average longitudinal changes of clinical symptoms, DAT imaging, and CSF protein levels among 4 phenotypes during follow‐up

Characteristics	Group I PD	Group II PD‐OD	Group III PD‐pRBD	Group IV PD‐DH	GEE *P* Value
Motor symptoms					
MDS‐UPDRS Part II	0^a^	1.2 (0.3–2.2)	1.7 (0.8–2.6)	2.2 (1.1–3.3)	***P* II = 0.009**
					***P* III <0.001**
					***P* IV <0.001**
MDS‐UPDRS Part III	0^a^	2.2 (−0.1 to 4.5)	1.6 (−0.2 to 3.4)	1.9 (0.09–3.8)	*P* II = 0.067
					*P* III = 0.089
					***P* IV = 0.040**
Nonmotor symptoms					
MDS‐UPDRS Part I	0^a^	0.8 (−0.2 to 1.6)	1.9 (1.0–2.7)	2.6 (1.7–3.5)	*P* II = 0.058
					***P* III <0.001**
					***P* IV <0.001**
MoCA	0^a^	−0.4 (−0.8 to 0.03)	−0.6 (−1.0 to −0.2)	−1.0 (−1.7 to −0.4)	*P* II = 0.068
					***P* III = 0.003**
					***P* IV = 0.001**
HVLT‐R total recall, totalscore	0^a^	−2.9 (−4.5 to −1.3)	−1.6 (−3.1 to −0.2)	−4.8 (−6.6 to −3.0)	***P* II <0.001**
					***P* III = 0.026**
					***P* IV <0.001**
HVLT‐R delayed recall	0^a^	−3.1 (−4.7 to −1.3)	−1.4 (−2.8 to −0.1)	−4.6 (−6.5 to −2.6)	***P* II <0.001**
					***P* III = 0.035**
					***P* IV <0.001**
HVLT‐R retention	0^a^	−2.1 (−3.8 to −0.4)	−1.5 (−2.9 to −0.1)	−2.7 (−4.7 to −0.6)	***P* II = 0.013**
					***P* III = 0.030**
					***P* IV = 0.009**
LNS scaled score	0^a^	−0.3 (−0.7 to 0.02)	−0.4 (−0.7 to −0.09)	−0.8 (−1.2 to −0.3)	*P* II = 0.066
					***P* III = 0.010**
					***P* IV = 0.001**
SDMT	0^a^	−1.7 (−3.1 to −0.4)	−1.9 (−3.3 to −0.6)	−3.2 (−4.8 to −1.7)	***P* II = 0.011**
					***P* III = 0.004**
					***P* IV <0.001**
SFT	0^a^	−0.7 (−2.3 to 0.8)	−0.74 (−1.8 to 0.9)	−0.5 (−2.3 to1.2)	*P* II = 0.370
					*P* III = 0.517
					*P* IV = 0.573
Scopa‐AUT	0^a^	0.1 (−0.7 to 1.0)	1.1 (0.2–1.9)	2.1 (1.0–3.3)	*P* II = 0.786
					***P* III = 0.009**
					***P* IV <0.001**
Gastrointestinal	0^a^	0.03 (−0.3 to 0.3)	0.5 (0.2–0.8)	0.6 (0.2–1.0)	*P* II = 0.836
					***P* III = 0.001**
					***P* IV = 0.001**
Urinary	0^a^	−0.2 (−0.6 to 0.1)	0.03 (−0.3 to 0.4)	0.6 (0.1–1.0)	*P* II = 0.298
					*P* III = 0.864
					***P* IV = 0.009**
Cardiovascular	0^a^	0.08 (−0.04 to 0.2)	0.2 (0.1–0.4)	0.3 (0.1–0.4)	*P* II = 0.199
					***P* III <0.001**
					***P* IV <0.001**
DAT imaging					
Low caudate	0^a^	0 (−0.04 to 0.04)	−0.05 (−0.1 to −0.003)	−0.1 (−0.1 to −0.04)	*P* II = 0.984
					***P* III = 0.038**
					***P* IV <0.001**
Low putamen	0^a^	−0.01 (−0.03 to 0.01)	−0.002 (−0.02 to 0.02)	−0.02 (−0.04 to −0.001)	*P* II = 0.372
					*P* III = 0.859
					***P* IV = 0.042**
Mean caudate	0^a^	−0.02 (−0.07 to 0.02)	−0.04 (−0.09 to 0.003)	−0.1 (−0.1 to −0.05)	*P* II = 0.277
					*P* III = 0.065
					***P* IV <0.001**
Mean putamen	0^a^	−0.01 (−0.04 to 0.007)	−0.01 (−0.03 to 0.01)	−0.02 (−0.05 to −0.004)	*P* II = 0.162
					*P* III = 0.367
					***P* IV = 0.023**
CSF, markers, pg/mL					
α‐syn	0^a^	−55 (−139 to 28)	−58 (−118 to 0.7)	−152 (−241 to −64)	*P* II = 0.193
					*P* III = 0.053
					***P* IV = 0.001**
Aβ42	0^a^	−33 (−71 to 4)	−34 (−71 to 2)	−53 (−95 to −10)	*P* II = 0.083
					*P* III = 0.065
					***P* IV = 0.014**
Tau	0^a^	−0.6 (−6.4 to 5.0)	−5.1 (−9.1 to 0.4)	−7.2 (−13.7 to 0.7)	*P* II = 0.814
					***P* III = 0.031**
					***P* IV = 0.028**
P‐tau	0^a^	−0.1 (−0.5 to 0.3)	−0.3 (−0.7 to 0.02)	−0.6 (−1.1 to −0.08)	*P* II = 0.665
					*P* III = 0.065
					***P* IV = 0.023**

Note: Data are provided as average change β coefficient (95% confidence interval).

^a^ Reference group.

Abbreviations: DAT, dopamine transporter; CSF, cerebrospinal fluid; PD, Parkinson's disease; OD, olfactory dysfunction; pRBD, probable rapid eye movement sleep behavior disorder; DH, dual hit; GEE, generalized estimating equation; MDS, Movement Disorder Society; UPDRS, Unified Parkinson's Disease Rating Scale; HVLT‐R, Hopkins Verbal Learning Test–Revised; LNS, Wechsler Memory Scale‐Third edition (WMS‐III) Letter Number Sequencing; SDMT, Symbol Digit Modalities Test; SFT, Semantic Verbal Language Fluency Test; Scopa‐AUT, Scales for Outcomes in Parkinson's Disease–Autonomic; α‐syn, α‐synuclein; Aβ42, β‐amyloid 1–42; P‐tau, phosphorylated tau at threonine 181.

Furthermore, group × time interactions in GEEs were applied to evaluate the differences in the clinical measurement scores among the 4 groups in a unit of time (year) during follow‐up, namely, the rates of progression (Table 4 and Table S1). As a result, the PD‐DH group showed faster progression in MDS‐UPDRS Part II (0.6 points/year faster, *P* < 0.001) and Part III (0.7 points/year faster, *P =* 0.029) than the PD group. The PD‐pRBD and PD‐OD groups also exhibited significantly faster progression in MDS‐UPDRS Part II than the PD group, but only the PD‐OD group had faster progression than the PD group in Part III (0.7 points/year faster, *P =* 0.037). Also, patients in the PD‐DH group had faster deterioration in nonmotor symptoms: MDS‐UPDRS Part I (0.5 points/year), MoCA (0.4 points/year), HVLT‐R total recall (1.0 points/year), LNS score (0.3 points/year), SDMT (1.4 points/year), Scopa‐AUT total score (0.5 points/year), and the cardiovascular domain of Scopa‐AUT (0.1 points/year), with all *P* values <0.05. Furthermore, Cox hazard survival analysis revealed that the PD‐DH subtype had a significantly higher MCI risk than the PD group (hazard ratio = 1.756, 95% confidence interval = 1.132–2.722, *P* = 0.012; Fig. S1).

**TABLE 4:** Longitudinal change rates of clinical symptoms, DAT imaging, and CSF protein levels among 4 phenotypes during follow‐up

Characteristics	Group I PD	Group II PD‐OD	Group III PD‐RBD	Group IV PD‐DH	GEE *P* Value
Motor symptoms					
MDS‐UPDRS Part II	0^a^	0.3 (0.09–0.6)	0.4 (0.1–0.6)	0.6 (0.3–0.8)	***P* II = 0.008**
					***P* III = 0.002**
					***P* IV <0.001**
MDS‐UPDRS Part III	0^a^	0.7 (0.04–1.3)	0.4 (−0.1 to 1.0)	0.7 (0.07–1.3)	***P* II = 0.037**
					*P* III = 0.125
					***P* IV = 0.029**
Nonmotor symptoms					
MDS‐UPDRS Part I	0^a^	0.1 (−0.1 to 0.3)	0.3 (0.1–0.6)	0.5 (0.3–0.8)	*P* II = 0.389
					***P* III = 0.005**
					***P* IV <0.001**
MoCA	0^a^	−0.07 (−0.2 to 0.07)	−0.2 (−0.4 to −0.09)	−0.4 (−0.6 to −0.1)	*P* II = 0.342
					***P* III = 0.002**
					***P* IV = 0.001**
HVLT‐R total recall, totalscore	0^a^	−0.7 (−1.4 to −0.1)	−0.4 (−1.0 to 0.1)	−1.0 (−1.7 to −0.3)	***P* II = 0.015**
					*P* III = 0.166
					***P* IV = 0.002**
HVLT‐R delayed recall	0^a^	−0.5 (−1.1 to 0.1)	−0.4(−1.0 to 0.08)	−1.4 (−2.2 to −0.7)	*P* II = 0.123
					*P* III = 0.097
					***P* IV <0.001**
HVLT‐R retention	0^a^	−0.1 (−0.9 to 0.6)	−0.3 (−1.0 to 0.2)	−1.5 (−2.4 to −0.7)	*P* II = 0.716
					*P* III = 0.240
					***P* IV <0.001**
LNS scaled score	0^a^	−0.1 (−0.2 to −0.02)	−0.07 (−1.0 to 0.05)	−0.3 (−0.4 to −0.1)	***P* II = 0.015**
					*P* III = 0.260
					***P* IV <0.001**
SDMT	0^a^	−0.7 (−1.1 to −0.3)	−0.7 (−1.3 to −0.2)	−1.4 (−2.1 to 0.8)	***P* II = 0.001**
					***P* III = 0.004**
					***P* IV <0.001**
SFT	0^a^	−0.5 (−1.0 to −0.01)	−0.4 (−0.9 to 0.09)	−0.5 (−1.1 to 0.1)	***P* II = 0.042**
					*P* III = 0.107
					*P* IV = 0.121
Scopa‐AUT	0^a^	0.03 (−0.2 to 0.3)	0.2 (−0.09 to 0.5)	0.5 (0.06–1.0)	*P* II = 0.823
					*P* III = 0.162
					***P* IV = 0.027**
Gastrointestinal	0^a^	−0.03(−0.1 to 0.1)	0.06 (−0.05 to 0.1)	0.06 (−0.08 to 0.2)	*P* II = 0.640
					*P* III = 0.267
					*P* IV = 0.385
Urinary	0^a^	−0.08(−0.2 to 0.05)	−0.01(−0.1 to 0.1)	−0.09(−0.1 to 0.3)	*P* II = 0.233
					*P* III = 0.837
					*P* IV = 0.416
Cardiovascular	0^a^	0.02 (−0.02 to 0.07)	0.06 (−0.003 to 0.1)	0.1 (0.03 to 0.1)	*P* II = 0.286
					*P* III = 0.061
					***P* IV = 0.007**
DAT imaging					
Low caudate	0^a^	0.01 (−0.01 to 0.04)	−0.01 (−0.04 to 0.009)	−0.03 (−0.06 to −0.006)	*P* II = 0.301
					*P* III = 0.192
					***P* IV = 0.019**
Low putamen	0^a^	0.004 (−0.01 to 0.01)	−0.006 (−0.01 to 0.007)	−0.007 (−0.02 to 0.005)	*P* II = 0.586
					*P* III = 0.346
					*P* IV = 0.247
Mean caudate	0^a^	0.009 (−0.01 to 0.03)	−0.01 (−0.04 to 0.007)	−0.03 (−0.06 to −0.008)	*P* II = 0.535
					*P* III = 0.167
					***P* IV = 0.012**
Mean putamen	0^a^	0.007 (−0.008 to 0.02)	−0.005 (−0.01 to 0.009)	0.000 (−0.01 to 0.01)	*P* II = 0.346
					*P* III = 0.445
					*P* IV = 0.983
CSF, markers, pg/mL					
α‐syn	0^a^	−19 (−78 to 39)	−35 (−84 to 13)	−77 (−149 to −5)	*P* II = 0.519
					*P* III = 0.157
					***P* IV = 0.034**
Aβ42	0^a^	−5 (−28 to 17)	−12 (−37 to 11)	−15 (−45 to 14)	*P* II = 0.643
					*P* III = 0.300
					*P* IV = 0.309
Tau	0^a^	1.2 (−2.6 to 5.2)	−2.3 (−5.4 to 0.7)	−3.3 (−6.8 to 0.06)	*P* II = 0.521
					*P* III = 0.144
					*P* IV = 0.055
P‐tau	0^a^	0.08 (−0.2 to 0.3)	−0.2 (−0.5 to 0.08)	−0.2 (−0.5 to 0.07)	*P* II = 0.569
					*P* III = 0.156
					*P* IV = 0.135

Note: Data are shown as change rate (points per year) β coefficient (95% confidence interval).

^a^ Reference group.

Abbreviations: DAT, dopamine transporter; CSF, cerebrospinal fluid; PD, Parkinson's disease; OD, olfactory dysfunction; RBD, rapid eye movement sleep behavior disorder; DH, dual hit; GEE, generalized estimating equation; MDS, Movement Disorder Society; UPDRS, Unified Parkinson's Disease Rating Scale; MoCA, Montreal Cognitive Assessment; HVLT‐R, Hopkins Verbal Learning Test–Revised; LNS, WMS‐III Letter Number Sequencing; SDMT, Symbol Digit Modalities Test; SFT, semantic verbal language fluency test; Scopa‐AUT, Scales for Outcomes in Parkinson's Disease–Autonomic; α‐syn, α‐synuclein; Aβ42, β‐amyloid 1–42; P‐tau, phosphorylated tau at threonine 181.

### Longitudinal Changes in DAT and CSF Protein Levels

The PD‐DH subtype was associated with a greater and faster decline in dopaminergic innervation of the caudate, as evidenced by SBR values on the more‐affected side and the means (0.1 more decline with *P* < 0.001 and 0.03 per year faster with *P* = 0.019; Tables 3 and 4). Moreover, the PD‐DH group showed a greater and faster decline in CSF α‐syn levels than the PD group (152 pg/mL lower with *P* = 0.001 and 77 pg/mL per year faster, *P* = 0.034; Tables 3 and 4). Furthermore, patients in the PD‐DH group had also significantly lower levels of CSF Aβ42, tau, and P‐tau levels (53 pg/mL, 7.2 pg/mL, and 0.6 pg/mL lower than the PD group, with *P* = 0.014, 0.028, and 0.023, respectively), but no difference was found in the decline rates (Tables 3 and 4).

## Discussion

In the present study, we found that the concurrent OD and pRBD, namely, the PD‐DH subtype, was associated with faster disease progression, especially in nonmotor symptoms, including cognitive deficits and autonomic dysfunctions. Moreover, the PD‐DH subtype also showed higher scores and faster progressions in motor symptom measurements, including the MDS‐UPDRS Part II and Part III scores, and was found to experience a more rapid decline in caudate DAT uptake and CSF α‐syn levels during follow‐up. These findings indicate that PD‐DH may be served as a more aggressive phenotype in PD.

Although previous studies have reported that pRBD and OD are likely risk factors for motor dysfunction in PD, their effect on motor progression is still controversial.^5^, ^6^, ^7^, ^11^, ^16^ Pagano et al subgrouped patients with PD‐pRBD based on SPECT‐DAT imaging and CSF pathological protein levels and found that the presence of pRBD was associated with faster progression of motor dysfunction only in patients with PD with greater α‐syn and dopaminergic pathology.^7^ In addition, in another cross‐sectional study, Rossi et al reported that patients with PD with OD did not exhibit faster progression in motor symptoms than those with normal olfactory function.^16^ In our study, we evaluated the relationship between disease progression and the presence of pRBD or OD in a population of patients with de novo PD, and our results suggested that the concurrence of pRBD and OD was associated with higher scores in MDS‐UPDRS Part II and Part III measurements during follow‐up compared with the PD group. It is worth noting that the average increments in the current study (2.2 and 1.9 points for MDS‐UPDRS Part II and Part III, respectively) seem to be below the suggested meaningful clinical changes (2.51 points for deterioration in MDS‐UPDRS Part II and 4.63 points in Part III), according to previous studies.^23^, ^24^ However, in the further analysis of group × time interactions in GEEs, significant faster progressions were also observed in MDS‐UPDRS Part II and Part III scores between the PD‐DH and PD groups, indicating that patients in the PD‐DH group are likely to deteriorate faster in motor symptoms, and further study with a longer follow‐up may help to confirm a clinical meaningful change.

The relationship between cognitive decline and pRBD or OD has been well studied, and both pRBD and OD were reported as risk factors for cognitive impairment in PD.^6^, ^11^, ^25^ However, a recent study indicated that the PD‐pRBD subgroup may vary in terms of progression of cognitive symptoms depending on CSF α‐syn and Aβ42 levels, showing that patients with PD with pRBD and greater α‐syn and Aβ42 pathology were prone to develop MCI.^7^ Also, in a 4.4‐year prospective study, Anang et al did not find any difference in cognitive decline progression between patients with PD with or without OD.^26^ These studies indicated the heterogeneous progression of cognitive decline in PD. Our study demonstrated that the concurrent presence of pRBD and OD may act synergistically in increasing the risk of MCI throughout the disease course, whereas the patients in the PD‐pRBD and PD‐OD groups developed only a moderate cognitive decline. The pathophysiological mechanism responsible for cognitive decline in PD‐DH is unclear, and the caudate nucleus may play an important role. The dopaminergic decline in the caudate is reportedly responsible for cognitive impairment.^25^, ^27^ In our study, only the PD‐DH subtype was associated with greater decline in dopaminergic innervation of the caudate, indicating that the caudate nucleus may be involved in the association between cognitive decline and PD‐DH. Last but not least, previous research demonstrated that in patients with PD with pRBD, greater pathological protein burden, measured as lower levels of CSF α‐syn or Aβ42, was related to a more rapid decline in cognitive performance.^7^ Interestingly, our findings indicated that the PD‐DH subtype was associated with a more rapid decrease of CSF α‐syn during follow‐up when compared with the PD‐pRBD or PD‐PD subgroup, in line with previous findings.

It has been previously reported that patients with PD with pRBD manifest more severe autonomic dysfunction.^7^, ^28^ Our findings are consistent with these observations, showing that patients in the PD‐pRBD and PD‐DH subgroups experienced greater autonomic dysfunction at baseline. Interestingly, De Pablo‐Fernandez et al found that early autonomic dysfunction predicts a poor PD prognosis.^29^ In our study, the PD‐DH group showed the most rapid progression in autonomic dysfunction among all groups and was also associated with faster progression in motor measurements and cognitive impairment. Our findings are in line with these observations and further highlight the importance of PD‐DH in autonomic dysfunction progression.

Only a few longitudinal studies have evaluated the relationship between stratum DAT reduction and pRBD in PD. Kim et al analyzed a population of de novo PD from the PPMI database and found that patients with PD with pRBD exhibited greater decline in dopaminergic innervation in the caudate during a 4‐year follow‐up than those without pRBD.^30^ In the present study, a significantly more rapid DAT reduction in the caudate was only observed in the PD‐DH group, but not in the PD‐pRBD group, when compared with the PD group. Furthermore, the relationship between CSF α‐syn levels and PD has been well studied. Most previous studies indicated a decrease of CSF α‐syn levels in patients with PD compared with healthy controls and a decreasing trend of CSF α‐syn levels during disease progression.^31^, ^32^, ^33^ Consistently, our findings demonstrated that PD‐DH was associated with a faster decrease of CSF α‐syn levels and a faster disease progression.

Our findings indicated that the concurrent presence of pRBD and OD was related to faster disease progression in PD, but the underlying pathological mechanisms are unclear. Based on the dual‐hit hypothesis, the olfactory bulb and dorsal motor nucleus of the vagus might be the main entry points of the α‐syn pathology that originates in the periphery.^34^, ^35^ The pathological propagation process underlying PD adopts a 2‐pronged attack: retrogradely via the enteric nervous system and dorsal motor nucleus of the vagus fibers, reaching the brainstem and eventually affecting the substantia nigra, where RBD and autonomic dysfunction are involved early in clinical manifestations; and anterogradely via olfactory pathways to the brain, of which OD could be a clinical marker.^35^, ^36^ In our study, the PD‐DH subtype has both pRBD and OD symptoms, indicating a dual hit of α‐syn pathology from both vagal and olfactory pathways to the brain, which may eventually lead to a faster disease progression as a result of a higher α‐syn burden. Further pathological animal studies and prospective cohorts may help to better understand the underlying mechanisms of the PD‐DH subtype.

Some limitations of this study should be noted. First, although GEEs can overcome the dropout data to optimally estimate the effect size, the sample size attenuation during follow‐up because of attrition may have introduced survival bias in the present study. Second, there was a difference in age and sex distribution among groups at baseline. Therefore, in the present study, we corrected age and sex as covariates in every statistical model and thus they would not bias the results. Moreover, the presence of pRBD was assessed by the RBD Screening Questionnaire, but not video polysomnography, which might increase misclassification in our study. Further studies with larger sample sizes and better study design are warranted to confirm our findings.

In conclusion, the concurrent presence of pRBD and OD may predispose toward a more aggressive phenotype in PD, characterized by faster progressions in motor measurements and nonmotor symptoms. In addition, the PD‐DH subtype was associated with a faster α‐syn decrease in the CSF and more severe dopaminergic dysfunction in the caudate. Thus, our findings suggest that screening patients with PD for pRBD and OD may help in assessing progression; PD‐DH could be considered a precise subtype for potential disease‐modifying therapy trials. Future research on the underlying pathophysiological mechanisms of PD‐DH is needed to confirm these findings.

## Author Roles

(1) Research Project: A. Conception, B. Organization, C. Execution; (2) Statistical Analysis: A. Design, B. Execution, C. Review and Critique; (3) Manuscript: A. Writing of the First Draft, B. Review and Critique.

J.‐L.P.: 1A, 1B, 2A, 2C, 3A, 3B

B.‐R.Z.: 1A, 1B, 2C, 3B

Y.C.: 1B, 1C, 2A, 2B, 2C, 3A, 3B

N.‐J.X.: 1C, 2A, 2B, 2C, 3A, 3B

Y.F.: 1C, 2C, 3B

C.‐Y.J.: 1C, 2C, 3B

Y.‐L.L.: 2C, 3B

J.T.: 2C, 3B

Y.‐P.Y.: 2C, 3B

X.‐Z.Y.: 2C, 3B

## Disclosures

**Ethical Compliance Statement:** Parkinson's Progression Markers Initiative Online is an observational study collecting participant reported information from people with and without Parkinson's Disease (NCT04477785). Written informed consent was obtained from all participants or their guardians. We confirm that we have read the Journal's position on issues involved in ethical publication and affirm that this work is consistent with those guidelines.

**Funding Sources and Conflicts of Interest:** This work was supported by grants from the National Natural Science Foundation of China (Grant 81771216), the Key Research and Development Program of Zhejiang Province (Grant 2020C03020), and the Natural Science Foundation of Zhejiang Provincial (No. LY18H090003). None of the authors have received any funding from any institution.

**Financial Disclosures for the Previous 12 Months:** The authors disclose no conflicts of interest, including personal relationships, interests, grants, employment, affiliations, patents, inventions, honoraria, consultancies, royalties, stock options/ownership, or expert testimony in the past 12 months.

## Supporting information


**Table S1.** Dual hit in PD (PD‐DH) group as reference group for longitudinal change rates in clinical symptoms, dopamine transporter imaging, and cerebrospinal fluid protein levels among four phenotypes during follow‐up
**Figure S1.** Survival curve of the impact of probable rapid eye movement sleep behavior disorder (pRBD) and olfactory dysfunction on mild cognitive impairment
