# Supplementary material for: Association of Concurrent Olfactory Dysfunction and Probable Rapid Eye Movement Sleep Behavior Disorder with Early Parkinson's Disease Progression
Source: Mov Disord Clin Pract. 2022 Jul 22;9(7):909–19. doi: 10.1002/mdc3.13511 (PMC9547146; doi:10.1002/mdc3.13511)

***Table S1***. PD-DH group as reference group for longitudinal change rates in clinical symptoms, DAT imaging and CSF protein levels among four phenotypes during follow up

| **Change rates (points per year)**  **β-coefficient (95% CI)** | **Group I**  **PD** | **Group II**  **PD-OD** | **Group III**  **PD-pRBD** | **Group IV**  **PD-DH** | **GEEs**  **P-value** |
| --- | --- | --- | --- | --- | --- |
| **Motor symptoms** | | | | | |
| MDS-UPDRS part II | -0.6(-0.8- -0.3) | -0.2(-0.6- 0.1) | -0.1(-0.5- 0.1) | 0^a^ | **pI<0.001**  pII=0.168  pIII=0.289 |
| MDS-UPDRS part III | -0.7(-1.3- -0.07) | -0.02(-0.8-0.7) | -0.2(-1.0- 0.4) | 0^a^ | **pI=0.029**  pII=0.953  pIII=0.425 |
| **Nonmotor symptoms** | | | | | |
| MDS-UPDRS part I | -0.5(-0.8- -0.3) | -0.4 (-0.7- -0.1) | -0.2(-0.4- 0.09) | 0^a^ | **pI<0.001**  **pII=0.001**  pIII=0.175 |
| MoCA | 0.4(0.1-0.6) | 0.3(0.07-0.6) | 0.1(-0.1-0.4) | 0^a^ | **pI=0.001**  **pII=0.012**  pIII=0.234 |
| HVLT-total recall (T-score) | 1.0(0.3-1.7) | 0.2(-0.5-1.0) | 0.6(-0.1-1.4) | 0^a^ | **pI=0.002**  pII=0.475  pIII=0.094 |
| Delayed recall | 1.4(0.7-2.2) | 0.9(0.1-1.8) | 1.0(0.2-1.8) | 0^a^ | **pI<0.001**  **pII=0.028**  **pIII=0.014** |
| Retention | 1.5(0.7-2.4) | 1.4(0.3-2.4) | 1.1(0.2-2.1) | 0^a^ | **pI< 0.001**  **pII=0.007**  **pIII=0.013** |
| Recognition | 0.3(-0.3-1.1) | 0.2(-0.6-1.1) | 0.5(-0.3-1.4) | 0^a^ | pI=0.344  pII=0.584  pIII=0.231 |
| LNS scaled score | 0.3(0.1-0.4) | 0.1(-0.01-0.3) | 0.2(0.06-0.4) | 0^a^ | **pI<0.001**  pII=0.070  **pIII=0.008** |
| BJLO | 0.1(-0.05-0.2) | 0.1(-0.05-0.3) | -0.01(-0.1-0.1) | 0^a^ | pI=0.190  pII=0.179  pIII=0.877 |
| SDMT | 1.4(0.8-2.1) | 0.7(0.06-1.4) | 0.7(-0.04-1.4) | 0^a^ | **pI<0.001**  **pII=0.032**  pIII=0.066 |
| SFT | 0.5(-0.1-1.1) | -0.05(-0.7-0.6) | 0.05(-0.6-0.7) | 0^a^ | pI=0.121  pII=0.893  pIII=0.878 |
| Scopa-AUT | -0.5(-1.0- -0.06) | -0.5(-1.0- 0.01) | -0.2(-0.8- 0.2) | 0^a^ | **pI=0.027**  pII=0.056  pIII=0.277 |
| **DAT imaging** | | | | | |
| Low caudate | 0.03(0.006-0.06) | 0.04(0.01-0.08) | 0.01(-0.01-0.04) | 0^a^ | **p I=0.019**  **p II= 0.004**  p III= 0.325 |
| Low putamen | 0.007(-0.005-0.02) | 0.01(-0.004-0.02) | 0.001(-0.01-0.01) | 0^a^ | p I=0.247  p II= 0.156  p III= 0.885 |
| Mean caudate | 0.03(0.008-0.06) | 0.04(0.01-0.07) | 0.01(-0.01-0.05) | 0^a^ | **p I=0.012**  **p II=0.010**  p III=0.256 |
| Mean putamen | 0.000(-0.01-0.01) | 0.007(-0.01-0.02) | -0.005(-0.02-0.01) | 0^a^ | p I=0.983  p II=0.406  p III=0.532 |
| **CSF, markers, pg/mL** | | | | | |
| α-syn | 77(5-149) | 58(-17-133) | 42(-26-111) | 0^a^ | **p I=0.034**  p II=0.130  p III=0.230 |
| Aβ42 | 15(-14-45) | 10(-19-40) | 2(-28-33) | 0^a^ | p I=0.309  p II=0.502  p III=0.864 |
| Tau | 3.3(-0.06-6.8) | 4.6(0.4-8.8) | 1.0(-2.4-4.5) | 0^a^ | p I=0.055  **p II=0.031**  p III=0.551 |
| P-tau | 0.2(-0.07-0.5) | 0.3(-0.006-0.6) | 0.03(-0.3-0.3) | 0^a^ | p I=0.131  p II=0.054  p III=0.833 |

^a^Reference group.

Abbreviations: PD: Parkinson disease; pRBD: probable REM sleep behavior disorder; OD: olfactory dysfunctions; UPDRS: Unified Parkinson’s Disease Rating Scale; MoCA: Montreal Cognitive Assessment; HVLT: Hopkins Verbal Learning Test-Revised; LNS: WMS-III Letter Number Sequencing; SDMT: Symbol Digit Modalities Test; SFT: semantic verbal language fluency test; BJLO: Benton Judgment of Line Orientation; SCOPA: Scales for Outcomes in Parkinson’s Disease–Autonomic; α-syn: α-synuclein; Aβ42: β-amyloid 1–42; P-tau: phosphorylated tau.

***Figure S1.*** Survival curve of the impact of possible REM sleep behavior disorder (pRBD) and olfactory dysfunction on mild cognitive impairment


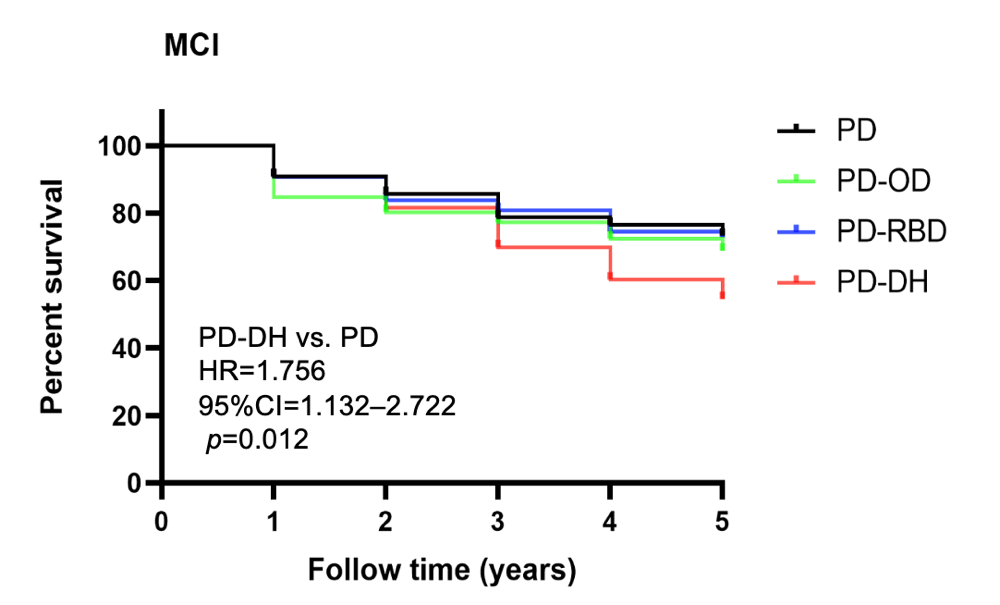

Supplement: Supplementary file 1 — Table S1. Dual hit in PD (PD‐DH) group as reference group for longitudinal change rates in clinical symptoms, dopamine transporter imaging, and cerebrospinal fluid protein levels among four phenotypes during follow‐up Figure S1. Survival curve of the impact of probable rapid eye movement sleep behavior disorder (pRBD) and olfactory dysfunction on mild cognitive impairment [file MDC3-9-909-s001.docx]
